# Metabolic status and lifestyle factors associated with gallbladder polyps: a covariance structure analysis

**DOI:** 10.1186/s12876-018-0882-z

**Published:** 2018-11-01

**Authors:** Song Leng, Ai Zhao, Qiang Li, Leilei Pei, Wei Zheng, Rui Liang, Hong Yan

**Affiliations:** 1 Department of Epidemiology and Health Statistics, School of Public Health, Xi’an Jiaotong University Health Science Center, Xi’an, China; 2 Health Management Center, The Second Hospital of Dalian Medical University, Dalian, China; 3 School of Public Health, Peking University Health Science Center, Beijing, China; 4 Division of Endocrinology and Metabolism, Department of obstetrics, Beijing Obstetrics and Gynecology Hospital, Capital Medical University, Beijing, China

**Keywords:** Gallbladder polyps, Blood lipid, Dietary intake, Body mass index, Metabolic status, Lifestyle factors

## Abstract

**Background:**

Gallbladder Polyps (GBP) are highly prevalent in China; however, the etiology of GBP has not been clearly defined. This study explored the associations between lifestyle factors and GBP and whether it mediated by metabolic factors or not.

**Methods:**

A total of 487 newly diagnosed GBP cases and 502 healthy controls were involved in this study. A questionnaire was used to investigate the socio-demographic characteristics and lifestyle factors. Food Intake Frequencies Questionnaire was used to obtain the food intake frequencies of seven food categories. Blood was tested for lipid profiles, fasting blood glucose and blood urine acid. A Covariance Structure Analysis was used in the analysis to explore the possible pathways between socio-demographic characteristics, lifestyle factors, metabolic factor and GBP.

**Results:**

The Covariance Structure Analysis showed that a higher BMI and elevated triglyceride level mediated the association between age and GBP. Lifestyle factors (smoking and drinking) and higher intake frequencies of fatty food (meat and viscera) also linked to higher BMI and higher triglyceride level, respectively, which were associated with GBP.

**Conclusion:**

In conclusion, age and lifestyle factors might be indirectly related with GBP through BMI and the triglyceride pathway.

## Background

Gallbladder Polyps (GBP) are defined as lesions protruding from the gallbladder mucosa and are one of the leading causes of hospital admissions related to gastrointestinal problems [1]. The presenting symptoms of GBP are nonspecific and vague, and in many cases, asymptomatic, which leads to a late diagnosis [2]. Although the reported rate of malignancy GBP is only 3–8% [2], it is a common public health issue in many countries, affecting millions of people and the prevalence is continuing to increase [3]. The prevalence of GBP is reported in the range 0.3% to 9.5% worldwide, depending on the studied population and the study design [4]. Previous studies reported that compared to western populations, Asian populations (mainly Japanese, Korean and Chinese) have a higher GBP prevalence and appear to be at a higher risk of gallbladder cancer [4–7]. Recently, the prevalence of GBP is estimated from 4 to 7% in different areas of China [4, 8].

To date, the etiology of GBP has not yet been clearly defined. Identifying risk factors for GBP will increase its understanding, diagnosis, and prevention. In China, HBV infection is highly prevalent, which is a strong risk factor for the development of GBP [4, 9]. The other reported risk factors associated with GBP mainly include unmodified socio-demographic factors such as age, gender, race, and family histories and modified lifestyle aspects, such as smoking, alcohol drinking, dietary habit and physical activity [4, 10]. According to previous studies, metabolic status such as obesity, hyperlipidemia, impaired glucose tolerance/diabetes and metabolic syndrome was strongly associated with GBP, however, there was paucity of the studies about the linkage between lifestyle factors and GBP [8, 10–12]. Therefore, it is important to explore whether these modifiable factors independently contribute to GBP or through metabolic disorder pathways.

In this study, both intermediate factors and lifestyle factors including dietary habit, smoking, alcohol use, and physical activities were investigated. The aims of this study are to determine the association between lifestyle factors and GBP and additionally to examine whether these associations were mediated by BMI or metabolic factors, using path analysis.

## Methods

### Participants

Study participants were enrolled at Second hospital of Dalian Medical University, China, from January 2016 to November 2016. In this period, a total of 806 patients were diagnosed as GBP with B mode ultrasound based on the ICD-10: K82.808. We excluded those 1) with previously diagnosed or self-reported GBP and other gallbladder and hepatic disease (includes different types of hepatitis), 2) with cancer, infectious disease or other severe disease(such as autoimmune disease), 3) with physical disability and 4) with mental disease and impaired memory. There were 501 patients who were eligible, with 487volunteering to participate in this study, and who completed the questionnaire and blood test.

The control group was taken from 600 healthy volunteers who underwent annual routine physical examination during the same period as the case group and determined without GBP according to B mode ultrasound results and medical records. Total of 580 of them were eligible for this study according to the same exclusion criteria as the case group. Finally, 502 of them volunteered to participant in this study and completed the questionnaire. The results of blood test were obtained from the participants’ routine physical examination with consent.

### Data collection

Data were collected from cases and controls by trained registered nurses using an interviewer-administered questionnaire with regard to socio-demographic characteristics and lifestyle factors. The preliminary questionnaire tests were completed prior to data collection.

Regular patterns of food consumption were assessed using Food Frequency Questionnaire; seven different kinds of food groups were investigated: 1) meat, 2) viscera, 3) fried food,4) vegetable, 5) fruits, 6) alcoholic drinks and 7)tea. Smoking was defined as daily smoking one or more cigarettes (or any other types of tobacco equals to 1 cigarette) and lasting for at least one year. The previous smoking was defined as who successfully quitted smoking over 1 year. Physical activity of participants attending in the past months was asked for the types (categorized as light, moderate, or vigorous physical activities), frequencies and duration.

Anthropometric measurements (height, weight and blood pressure) were performed for each participant. Fasting serum blood samples(fasting over 8 h) were collected by trained nurse in the morning and tested for lipid profiles (total cholesterol (TC), total triglyceride (TG), high density lipoprotein cholesterol (HDL-C) and low density lipoprotein cholesterol (LDL-C), fasting blood glucose(FBG) and blood uric acid (BUA)).

### Statistical analyses

SAS version 9.3 (SAS Institute, Inc., Cary, NC,USA) was used for statistical analysis. Data were presented as mean ± SD or percentage. Univariate analysis was performed to compare characteristics between case and control groups with Chi-square analysis or Independent T Test. Then, the crude associations were obtained by the method of logistic regression.

Covariance Structure Analysis was constructed to examine the pathways between predictors and GBP in AMOS 7.0(SPSS, Inc., Chicago, IL, USA). The outcome variable was GBP (binary variable). The independent variables were determined according to the univariate analysis (variables for which *P* < 0.1) which included age, lifestyle factors (smoking) and several dietary factors. The predictors which were highly relevant to smoking and meat intake (including drinking and viscera intake) were also involved in the model. The mediators were BMI and lipids profiles. The associations between the independent variables and the pathways that linked the independent variables to the GBP were determined by Structural Equation Model.

## Results

### Socio-demographic characteristics of participants

A total of 487 cases (292 men and 195 women) and 502 controls (275 men and 227 women) participated in this study. The majority ethnic group is Han(93.7%). The comparisons of socio-demographic characteristics were shown in Table 1. The case group had a higher age. There were no significant associations between gender, GBP and education level or family income.

**Table 1:** Comparisons of socio-demographic characteristics between case and control

Variables	Case	Control	*P*	Crud OR (95%CI)
Age(years)	50.6 ± 14.0	46.1 ± 12.7	< 0.001	1.03 (1.02,1.04)
Gender				
Male	292 (51.5)	275 (48.5)	0.100	Ref.
Female	195 (46.2)	227 (53.8)		0.81 (0.63,1.04)
Education level				
Senior high school or under	88 (47.6)	97 (52.4)	0.603	Ref.
Bachelor degree or above	399 (49.7)	408 (50.3)		1.09 (0.79,1.50)
Family average Monthly income (RMB: yuan)				
< 2000	23 (5.4)	27 (6.6)	0.207	Ref.
2000~ 3999	121 (28.3)	95 (23.2)		1.50 (0.81,2.72)
≥4000	283 (66.3)	288 (70.2)		1.15 (0.65,2.06)

Ref. The reference group

Binary logistic regression was used to obtain the crud odds ratios *OR* and its 95% CI

### Univariate analysis of lifestyle and dietary factors

According to the results of the univariate analysis, the current smokers and the ones with a higher intake frequencies of meat were significantly associated with GBP (Table 2). Physical activities, other types of food, alcoholic drinks and tea were not associated with GBP.

**Table 2:** Lifestyle and dietary factors between case and control groups

Variables		Case	Control	*P*	Crud OR (95%CI)
Smoking	Non-smoker	349 (71.7)	382 (76.1)	0.006	Ref.
	Current smoker	120 (24.6)	88 (17.5)		1.49 (1.09,2.04)
Physical activities	Previous smoker	18 (3.7)	32 (6.4)		0.62 (0.34,1.11)
	< 120 min/week	323 (90.5)	301 (93.2)	0.353	Ref.
	120~ 180 min/week	30 (8.4)	18 (5.6)		1.55 (0.85,2.84)
	> 180 min/week	4 (1.1)	4 (1.2)		0.93 (0.23,3.76)
Food intake frequencies(times/week)					
Meat	Never	159 (32.6)	229 (45.6)	< 0.001	Ref.
	1~ 2	215 (44.1)	209 (41.6)		1.48 (1.12,1.96)
	3~ 4	81 (16.6)	51 (10.2)		2.29 (1.53,3.43)
	≥5	32 (6.6)	13 (2.6)		3.55 (1.80,6.97)
Viscera	Never	225 (46.2)	273 (54.4)	0.207	Ref.
	1~ 2	223 (47.8)	206 (41.0)		1.37 (1.06,1.78)
	3~ 4	22 (4.9)	16 (3.2)		1.82 (0.94,3.51)
	≥5	5 (1.0)	7 (1.4)		0.87 (0.27,2.77)
Fried food	Never	143 (29.4)	184 (36.7)	0.109	Ref.
	1~ 2	265 (54.4)	247 (49.2)		1.38 (1.04,1.83)
	3~ 4	66 (13.6)	58 (11.6)		1.46 (0.97,2.22)
	≥5	13 (2.7)	13 (2.6)		1.29 (0.58,2.86)
Vegetables	Never	13 (2.7)	12 (2.4)	0.550	Ref.
	1~ 2	53 (10.9)	48 (9.6)		1.08 (0.49,2.41)
	3~ 4	97 (19.9)	118 (23.5)		1.10 (0.73,1.68)
	≥5	324 (66.5)	324 (64.5)		0.82 (0.60,1.12)
Fruits	Never	42 (8.6)	35 (7.0)	0.601	Ref.
	1~ 2	122 (25.1)	121 (24.1)		0.84 (0.50,1.41)
	3~ 4	108 (22.2)	147 (29.3)		0.61 (0.37,1.02)
	≥5	215 (44.1)	199 (39.6)		0.90 (0.55,1.47)
Alcohol drink	< 1	434 (89.0)	447 (89.0)	0.971	Ref.
	≥1	53 (10.9)	55 (11.0)		0.97 (0.67,1.48)
Tea	< 1	326 (66.9)	340 (67.7)	0.791	Ref.
	≥1	161 (33.1)	162 (32.3)		1.04 (0.80,1.35)

Ref. The reference group

Binary logistic regression was used to obtain the crud odds ratios *OR* and its 95% CI

### Univariate analysis of health indicators

Comparing health indicators between case and control groups, the case group had a significantly higher TG level. The BMI, blood pressure level, other lipid profiles, FBG and BUA were not associated with GBP(Table 3).

**Table 3:** Metabolic indicators between case and control groups

Variables	Case	Control	P	Crud OR (95%CI)
BMI (kg/m^2^)	24.6 ± 3.3	24.5 ± 3.8	0.763	1.01(0.97,1.04)
Blood pressure				
Diastolic pressure	127.1 ± 16.1	126.2 ± 17.4	0.404	1.00(1.00,1.01)
Systolic pressure	78.0 ± 11.6	77.7 ± 11.9	0.651	1.00(0.99,1.01)
Lipid profiles				
TC(mmol/L)	4.84 ± 0.94	4.89 ± 0.92	0.385	0.94(0.82,1.08)
TG(mmol/L)	1.51 ± 1.10	1.37 ± 0.81	0.028	1.16(1.02,1.33)
HDL-C(mmol/L)	1.27 ± 0.33	1.31 ± 0.31	0.063	0.69(0.47,1.02)
LDL-C(mmol/L)	2.62 ± 0.72	2.58 ± 0.66	0.360	1.09(0.91,1.31)
FBG (mmol/L)	5.7 ± 1.4	5.7 ± 2.4	0.820	0.99(0.93,1.06)
BUA (μmol/L)	339.0 ± 92.5	341.9 ± 96.5	0.633	1.00(1.00,1.00)

Binary logistic regression was used to obtain the crud odds ratios *OR* and its 95% CI

### Pathway analysis

**Fig. 1:**
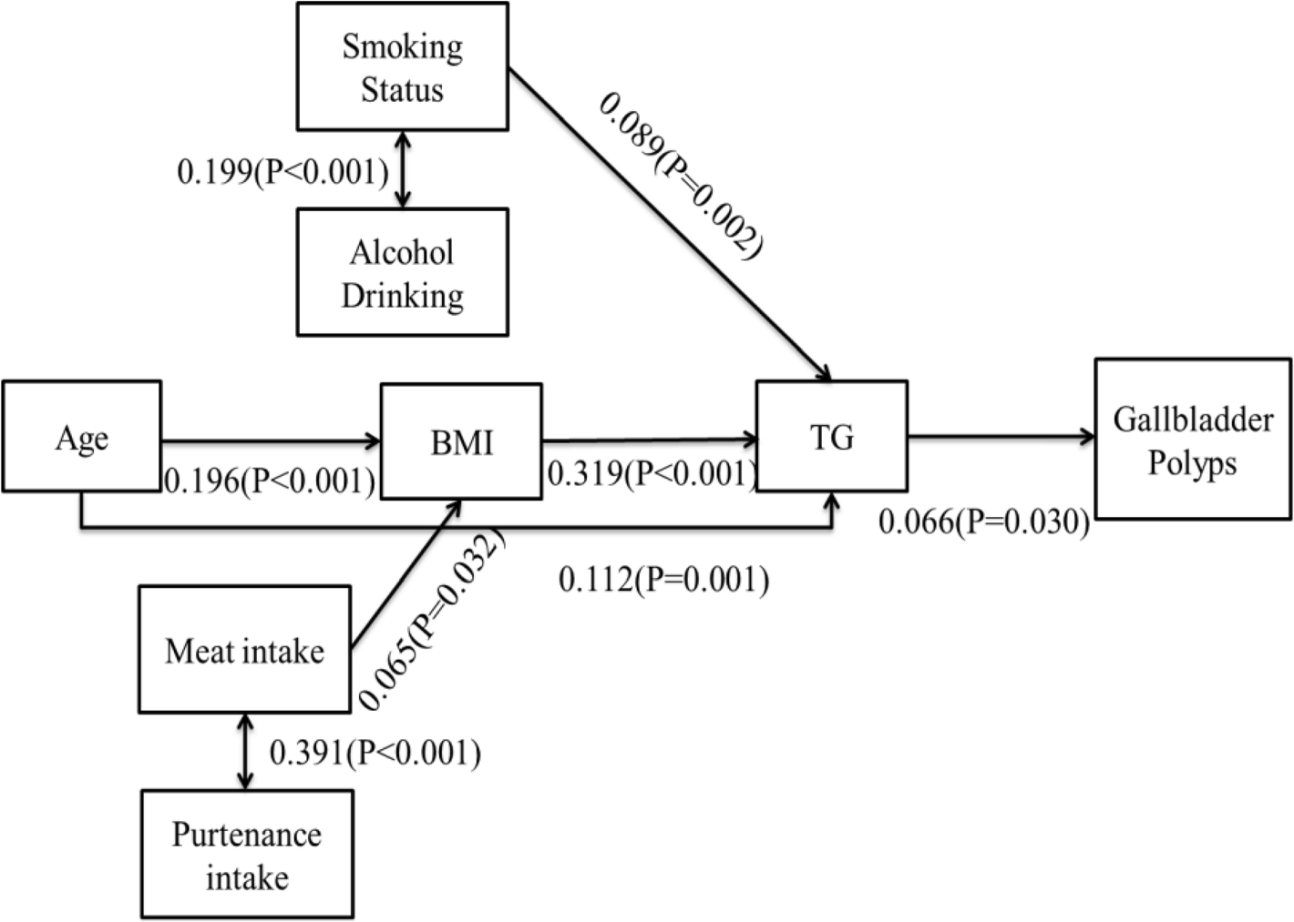
Standardized pathway between lifestyle and dietary factors and Gallbladder Polyps(Pathways were determined with exploratory methods, the goodness of fit index was 0.96, the comparative fit index was 0.93, and the root mean square error of approximation was 0.04)

The Structural Equation Model fit in the pathway analyses was adequate (The goodness of fit index (AGFI) was 0.96, comparative fit index (CFI) was 0.93, and the root mean square error of approximation was 0.04). The pathways determined in this study are depicted in Fig. 1. According to the Covariance Structure Analysis, elder and higher intake frequencies of meat are associated with the higher BMI; higher BMI linked to increased TG level; then the TG level is positively associated with GBP. In addition, age is also related to TG level. Meanwhile, the intake frequency of meat was highly correlated with the intake frequency of viscera, and these two predictors contribute to BMI. Smoking and drinking were also relevant, and were associated with TG.

## Discussion

This study was focused on exploring the risk factors for GBP in the population of China, especially focused on the modifiable lifestyle factors. The current study has supported and extended previous findings by demonstrating that dyslipidemia is associated with GBP. In addition, through pathway analysis, lifestyle factors might indirectly contribute to GBP through increasing BMI and the TG level pathway.

### Metabolic status with GBP

The etiology of GBP has not been clearly defined, the risk factors might be different among different types of GBP. In this study, we did not identify types of GBP; however, based on the previous study, the cholesterol polyps are the most common type (> 70%) [2]. Plenty of studies found that the abnormal metabolic status was associated with GBP. In Khairy’s study of 74 patients with gallbladder cholesterol polyps, 85.1% had dyslipidemia [13]. Considering different lipid profiles, several studies found that HDL-C level was negatively associated with GBP, while LDL-C level was positively related with GBP; however, the study results were inconsistent [2, 4, 14–16]. The roles of TG and TC on GBP were not clear. In this study, we found that TG level in the case group was significantly higher than in the controls. One Korean study also found similar results, where the elevated TG level was significantly associated with GBP [3]. In addition, abnormal TG level was reported to be associated with other gallbladder diseases, such as gallstones and gallbladder cancer, which might share a similar pathogenesis with GBP [17, 18]. In the hypothesis, some researchers suggested that the direct deposition of bile or blood cholesterol might contribute to the formation of cholesterol polyps; others inferred the alterations in hepatic cholesterol metabolism and altered mucosal esterification of free sterols from bile could contribute to the development of cholesterolemia [19].

There are conflicting results about the relationship between GBP and obesity; some studies found that increasing BMI or obesity status is associated with GBP, while Cantürk et al. Conducted a study on 432 patients and found that patients with GBP were not severely obese(BMI > 30) [8, 20, 21]. However, it seems that most studies agree with the formation of GBP being associated with fat metabolism [21–23]. In this study, in univariate analysis, no strong and direct association was found between BMI and GBP (*P* = 0.10); however, based on pathway analysis, high BMI is one of the important predictors which contribute to GBP through increasing the TG level pathway. This result indicated that abnormal fat metabolism is a risk factor of GBP. In addition, a higher age was reported as a risk factor of GBP in many previous studies [4, 8]. Based on the current results, there might be a possibility that BMI and TG increase with age and contribute to GBP.

In the current study, the impaired blood glucose and abnormal blood pressures were not related to GBP; these findings have been reported in some studies [24], but not in others [4, 9].Further prospective investigations are still needed to clarify the roles of metabolic disorders on GBP. In addition, recent studies showed that the abnormal metabolism not only contribute to the formation of GBP, but are also related to the polyps’ malignant transformation [25, 26]. Studies on exploring the metabolism effects of polyp transformation are also expected.

### Lifestyle factors with GBP

With regard to smoking, several previous studies found that this was inversely related to GB polyps; however, a Chinese study failed to find any association [1, 27, 28]. In our study, we found that current smoking was positively associated with GBP in univariate analysis. Another Chinese study also found smoking to be positively associated with GBP in univariate analysis; however, it was ruled out from multivariate logistic regression model [4]. We could not clarify the mechanism of smoking involved in GBP formation; however, according to the results of pathway analysis, smoking might contribute to GBP by elevating the TG level pathway. Drinking was found to be highly relevant to smoking in this study, and might combine with smoking, contributing to increase TG to GBP pathway. On the contrary, one animal experimental study reported that alcohol reduces biliary cholesterol saturation and increases the serum levels of high-density lipoprotein [29]. Similarly, one population-based study found a protective effect of alcohol use on GBP [27]; however, other study results failed to find this relationship [4]. The inconsistent results might be due to the different study design and different types of alcoholic drink. As symbols of lifestyle, there are many factors between smoking/drinking and GBP; further studies including etiological studies are necessary to clarify the roles of smoking and drinking in GBP.

Lots of studies have focused on dietary effects of gallbladder stones or gallbladder cancer, but not GBP. High fat intake seems to contribute to many gallbladder diseases [30]. For GBP, one Korean study reported GBP tended to be less common in vegetarians than in controls; however, the difference was statistically insignificant [31]. In this study, we found a higher frequency of meat intake associated with GBP in univariate analysis, and through pathway analysis, fatty food was related to a higher BMI and resulted in a high TG level associated with GBP. We inferred that excessive fatty food intake might result in the imbalances and increased plasma cholesterol concentration and/or induced hepatic hypersecretion of biliary cholesterol which causes GBP. More studies on the dietary effects of GBP are needed to fill in the gaps regarding dietary recommendations to prevent gallbladder disease.

#### Limitation

With a case-control design, inherent limitations of this study were unavoidable and the results should be treated with caution. The causality between risk factors and GBP in this study could not been observed. Although the cases were all newly diagnosed, recall bias might exist and residual confounding by imprecisely measured or unmeasured factors remains possible for our findings.

In this study, we did not identify types of GBP, the risk factors and pathological pathways might be different for different types of GBP.

## Conclusion

This study first use the Covariance Structure Analysis to explore the pathway among lifestyle factors, metabolic status and GBP. The current findings demonstrated that dyslipidemia is highly associated with GBP. More importantly, several modifiable factors such as smoking and high intake frequencies of meat or viscera might be the initial risk factors for GBP formation through increasing BMI and TG level pathways. More studies exploring modifiable factors with GBP are needed to build a strategy to prevent GBP.

## Abbreviations

BUA: Blood uric acid
FBG: Fasting blood glucose
GBP: Gallbladder polyps
HDL-C: High density lipoprotein cholesterol
LDL-C: Low density lipoprotein cholesterol
TC: Total cholesterol
TG: Total triglyceride cholesterol
